# Large pedunculated colonic lipoma: a rare cause of colorectal intussusception in adults

**DOI:** 10.11604/pamj.2020.36.200.24606

**Published:** 2020-07-21

**Authors:** Hakim Zenaidi, Imen Ben Ismail, Fatma Rekik, Mouelhi Aziz, Saber Rebii, Ayoub Zoghlami

**Affiliations:** 1 Department of General Surgery, Trauma and Burns Center, Ben Arous, Tunisia

**Keywords:** Lipoma, intussusception, bowel obstruction, colorectal resection

## Abstract

Colo-rectal intussusception is rare in adults and is often secondary to malignant lesions, rarely benign lesions such as colonic lipomas can also be the cause. We present the case a 60-year-old man who presented to the emergency department with acute abdominal pain. On physical examination, the abdomen was distended with diffuse tenderness. CT scan of the abdomen revealed a colo-rectal intussusception secondary to a rectal lipoma with parietal pneumatosis of the invaginated loop. An emergency laparotomy was performed. Intraoperatively the radiological findings were confirmed. A rectosigmoid resection (Hartmann's procedure) taking off the lipoma and the invaginated segment of the colon was performed and the patient had an unevent full recovery. Histopathology confirmed a 6cm sub-mucosal lipoma without evidence of malignancy. As the diagnosis of a benign disease in patients presenting with colonic intussusception can only be made on pathological examination, this entity should be managed as a malignant lesion due to the high incidence of malignancy.

## Introduction

Intussusception is a rare clinical entity in adults, defined as the telescoping of a proximal segment of a gastrointestinal tract intoan adjacent distal segment [1]. It accounts for 1-3% of all cases of bowel obstruction [2]. Colonic lipoma is rare and can serve as the lead point for this intussusception [3]. We report the case of a giant colonic lipoma causing a colorectal intussusception complicated with an acute bowel obstruction.

## Patient and observation

**Figure 1:**
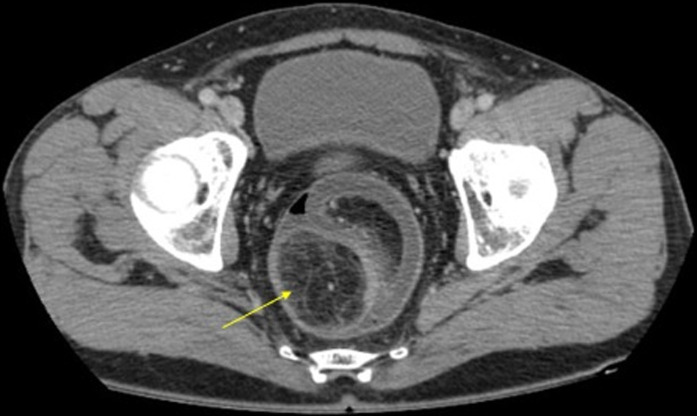
axial view of a CT scan of the abdomen showing a lipoma of 6cm of diameter filling the rectum and being the lead point to a colorectal intussusception (yellow arrow)

**Figure 2:**
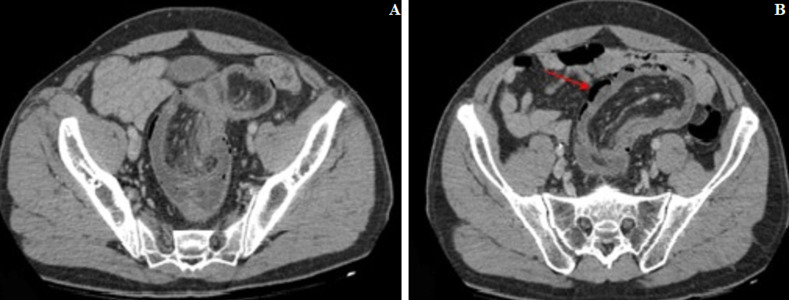
axial view of a CT scan of the abdomen showing the colo-rectal intussusception (A) with invaginated mesenteric fat and parietal pneumatosis (redarrow) (B)

**Figure 3:**
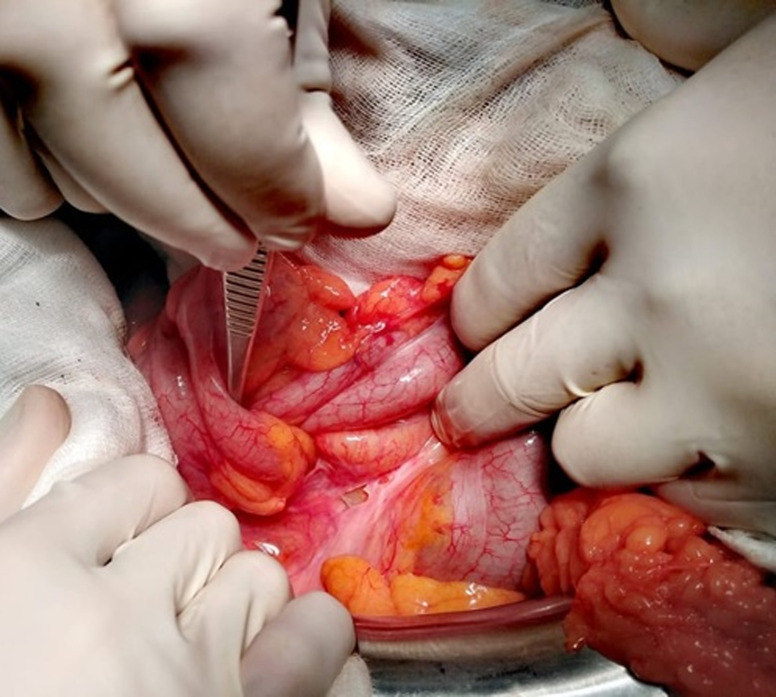
intraoperative view of the colo-rectal intussusception

**Figure 4:**
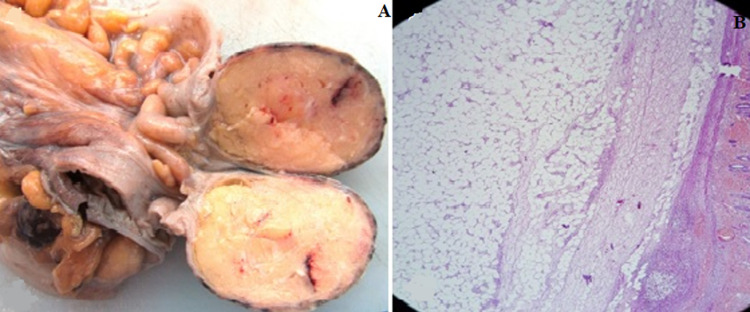
(A) gross examination showing a round pedunculated colonic lipoma measuring 6cm; (B) microscopic examination revealing fat cells proliferating in the submucosal layer, with inflammatory and ischemic changes

A 60-year-old man, with no medical or surgical history, presented to the emergency department with acute abdominal pain. He reported a short history of constipation and intermittent bleeding through the rectum for the last two weeks. On physical examination, vital signs were normal. The patient was afebrile. The abdomen was distended with diffuse tenderness. Furthermore, no palpable abdominal mass was detected. Rectal examination revealed a smooth and well-circumscribed mass lying in 3cm from the anal verge. Laboratory investigations on admission were normal except for a high level of weight blood cells (WBC = 19000 C/ml) and C-reactive protein (CRP = 170 mg/l). Computed tomography scan (CT) of the abdomen showed a 6cm soft-tissue mass with fat density within the rectum likely to be the lead point for a colorectal intussusception (Figure 1). Large bowel loops were dilated with the presence of parietal pneumatosis (Figure 2). The diagnosis of intussusception due to a lipomatous colonic tumor complicated with acute intestinal obstruction was carried out. Given the presence of signs of occlusion and bowel wall compromise, an emergency laparotomy was performed revealing a large bowel distension preceding a colo-rectal intussusception (Figure 3). An attempt of a manual desinvagination was unsuccessful. A rectosigmoid resection including the lipoma and the invaginated segment of the colon was performed. Due to colon dilatation, we decided not to perform an anastomosis and Hartmann´s procedure was considered to be more cautious. The postoperative recovery was uneventful and the patient was discharged on the fourth postoperative day. Gross examination of the resected specimen showed the presence of a round pedunculated colonic lipoma measuring 6cm (Figure 4). Microscopic examination revealed fat cells proliferating in the submucosal layer, with inflammatory and ischemic changes and without any evidence of malignancy (Figure 4). The diagnosis of a colonic lipoma was then confirmed.

## Discussion

Intussusception is more commonly seen in pediatric patients and represents less than 5% of intestinal obstructions in adult [4]. The mean age is 54.4 years and the male-to-female ratio is 1: 1.3 [5]. This entity is described as an internal prolapse of the proximal bowel with its mesenteric into the lumen of a distal segment. The mechanism of intussusception usually involves a pathologic lesion in the bowel wall or mural abnormalities that reduce the normal peristaltism and serves as a lead point, generating an invagination of one segment of the bowel into the other [6]. The lead point may be absent in 8-20% of cases and the intussusception is then classified as idiopathic or primary [7]. Typically small intestine intussusception is secondary to benign lesions such as benign neoplasms, Meckel´s diverticuli, inflammatory lesions and postoperative adhesions and in only 25% it is due to malignant lesions [5]. On the other hand, large bowel intussusception is more commonly caused by malignant lesions which represents up to 66% of the cases [8]. According to their locations intussusceptions have been classified into: (1) entero-enteric, involving the small intestine; (2) colo-colic, involving the large intestine; (3) ileo-colic, involving the terminal ileum and ascending colon; and (4) ileo-cecal, involving the ileo-cecal valve as the lead point. For more accuracy, two other categories exists and may be included: (1) colo-rectal, the colon invaginates through rectal ampulla and (2) recto-rectal with rectum invaginating into the rectum with no anal protrusion [2].

Lipoma is a begnin tumor of a mesenchymal origin, it occurs more frequently in the large intestine, mostly in the right colon [9]. The incidence of colonic lipoma ranges from 0.2% to 4.4% and it occurs in order of decreasing incidence, in the cecum, ascending colon and sigmoid colon [10]. Colonic lipomas are usually asymptomatic and diagnosed incidentally during a colonoscopy or intraoperatively. They become symptomatic in 75% of cases when their size is larger than 4cm and present with non-specific symptoms such as abdominal pain, constipation, bleeding, perforation and obstruction [11]. The clinical presentation of intussusception in adults is non-specific and most cases present with a chronic or sub-acute symptoms that are suggestive of chronic constipation [7, 8, 12]. The acute forms are rare and represent less than 20% [2]. The classic pediatric triad presentation of intussusception (abdominal pain, bloody diarrhoea and palpable abdominal mass) is rare in adults and is present in only 10% of cases [9]. Symptoms can be either acute or chronic and abdominal pain is the most frequent clinical finding, followed by nausea and vomiting in 40-60% of the cases and rectal bleeding in 4-33% of cases [4, 13]. The duration of symptoms is longer in enteric and benign lesions than in malignant and colonic ones [13]. In our case, intussusception of the sigmoid colon into the rectum is was secondary to a colonic lipoma, however a review of the literature demonstrated that, other lesions in the sigmoid wall are more likely to cause intussusception, such as sigmoid polyp [11], a sigmoid adenocarcinoma [14, 15] and a sigmoid liposarcoma [12].

Abdominal computed tomography (CT) scan is the most specific diagnostic test for intussusception and is superior to ultrasonography and endoscopy [16]. Its sensitivity ranges from 71.4% to 87.5%, with a specificity neighboring 100% [9]. The characteristics of intussusception on CT are the ‘target’ or ‘sausage-shaped’ soft tissue mass, mesenteric vessels within the bowel lumen are also typical [6, 13, 14]. Moreover, complications of intussusception such as acute obstruction or bowel ischemia and etiologies of intussusception can also be diagnosed by CT. Flexible endoscopy of the lower gastro intestinal tract is a helpful diagnostic tool since it may determine the localisation of intussusception by detecting the underlying lesion serving as a lead point, in addition to that it represents a therapeutic option [8]. It is advisable only in individuals presenting chronic or sub-acute symptoms. Surgical resection is the recommended treatment when lipomas are complicated by intussusception or bowel obstruction [6, 11, 13]. Reduction of the intussusception is not recommended in the presence of inflammation or ischemia of the bowel wall or when a malignancy is suspected because of the high risk of perforation and dissemination of malignant cells [6, 15]. Small intestinal lesions are mostly begnin; reduction can be attempt if there are no signs of bowel ischemia, in order to prevent the development of short bowel syndrome [7]. Intussusception´s prognosis depends on the causative lesion´s nature and mortality increases from 8.7% for the begnin lesions to 52.4% for the malignant ones [5].

## Conclusion

Sigmoido-rectal intussusception in adults secondary to a colonic lipoma is rare. The diagnosis of intussusception in adults is challenging because its non specific symptoms. CT scan has been reported to be the most specific radiological investigation for the diagnosis of intussusception especially in acute forms. Surgical resection, is recommended because of the high probability of malignancy.
